# Supplementary material for: Association of DNA Promoter Methylation and BRAF Mutation in Thyroid Cancer
Source: Curr Oncol. 2023 Mar 2;30(3):2978–96. doi: 10.3390/curroncol30030227 (PMC10047424; doi:10.3390/curroncol30030227)
Supplement: Supplementary file 1 [file curroncol-30-00227-s001.zip › Figure_S1.pdf]

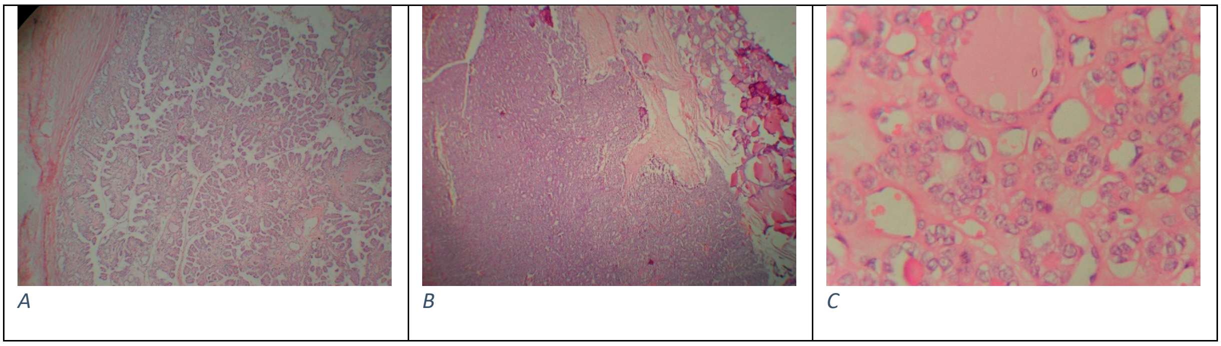

Figure S1. Photomicrograph of Thyroid carcinoma. (A) Papillary Thyroid Carcinoma (PTC); (B) Follicular Thyroid Carcinoma (FTC) and (C) Follicular Variant of Papillary Thyroid Carcinoma (FVPTC)
